# Phonotactic structure modulates the role of consonants and vowels in lexical processing: evidence from Spanish

**DOI:** 10.3389/fpsyg.2026.1757864

**Published:** 2026-05-18

**Authors:** María Rodríguez-Moreno, Amanda Flores, Paola Escudero, P. Javier López-Pérez, Juan L. Luque

**Affiliations:** 1 Neuroscience and Education Laboratory Leeduca, Department of Developmental and Educational Psychology, Faculty of Psychology and Speech Therapy, University of Málaga, Málaga, Spain; 2 Neuroscience and Education Laboratory Leeduca, Department of Basic Psychology, Faculty of Psychology and Speech Therapy, University of Málaga, Málaga, Spain; 3 The MARCS Institute for Brain, Behavior and Development, Western Sydney University, Westmead, NSW, Australia; 4 Centro Médico Cognitivo e Investigación (CMCI), Barranquilla, Colombia

**Keywords:** consonants, cross-situational statistical learning, minimal phonological pairs, phonological biases, vowels, word learning

## Abstract

**Introduction:**

Numerous studies have shown that consonants play a more crucial role than vowels in lexical recognition and learning, a phenomenon known as the C-Bias or C-advantage. While traditionally considered universal, recent findings suggest it may depend on language-specific phonotactic properties. This study examines whether the C-Bias is modulated by phonotactic constraints in Spanish, where vowels may have a discriminative advantage due to their distinctiveness.

**Methods:**

Seventy-seven native Spanish-speaking university students completed a Cross-Situational Word Learning task with pseudowords in two syllabic structures: monosyllabic (CVC) and disyllabic (CVCV).

**Results:**

The results revealed a vowel bias in monosyllabic pseudowords and a partial consonant bias in disyllabic pseudowords.

**Discussion:**

These findings indicate that phonotactic structure shapes the relative salience of consonants and vowels in lexical processing, challenging the assumption of a universal C-Bias. This study highlights the role of language-specific phonological constraints in word learning and suggests that phonological processing is more flexible than previously assumed.

## 1 Introduction

Traditionally, an asymmetry in phonological processing between consonants (Cs) and vowels (*Vs*) has been widely observed across various spoken language tasks. This phenomenon, known as the “C-Bias” or “C-advantage,” manifests when Cs appear to facilitate lexical processing to a greater extent than *Vs* (Nazzi and Cutler, 2019). This advantage has been documented in tasks such as word reconstruction (WR), novel word learning (WL), lexical decision-making, and speech segmentation in artificial languages (Creel et al., 2006; Cutler et al., 2000; Marks et al., 2002; Mulak et al., 2019; Toro et al., 2008; Van Ooijen, 1996; Zou et al., 2022). While most prior research has focused on spoken words, this C advantage has also been observed in written word processing (Carreiras et al., 2009; Escudero et al., 2023; Moates and Marks, 2012; New et al., 2008) and even under acoustically degraded listening conditions (De la Cruz-Pavía et al., 2023). Despite the robust evidence supporting the C-Bias across languages and tasks, recent studies in Cantonese and Mandarin have failed to find this advantage (Poltrock et al., 2018; Wiener and Turnbull, 2015), raising questions about its universality. In this study, we examine how the specific phonotactic properties of Spanish influence the manifestation of the C-Bias, proposing that manipulating these contexts can provide new insights into the interrelation between phonotactics and the C-Bias.

Robust evidence for C-Bias is consistently observed across various lexical processing tasks in different languages. In WL tasks, participants recall words that differ by a C more easily than those differing by a V (Mulak et al., 2019; Havy et al., 2014). For instance, Escudero et al. (2016) demonstrated this advantage in a Cross-Situational Word Learning (CSWL) paradigm. Participants learned pairs of pseudowords (e.g., *BON-TON* and *DEET-DET*) and novel objects, finding it harder to distinguish pairs differing by a V than by a C, suggesting a stronger influence of Cs in lexical learning. Similarly, the C advantage has been consistently observed in word reconstruction (WR) tasks, which involve presenting pseudowords that can become real words by changing a single phoneme (Van Ooijen, 1996; Cutler et al., 2000). In these tasks, the C advantage is demonstrated by participants taking less time to reconstruct words when they preserve all the Cs of the target pseudoword compared to when they preserve its Vs. For example, preserving Cs in “*shevel*” to form “*shovel*” requires less effort than changing a C to form “*level*.” According to these authors, this behavior is due to preserving Cs being more effective in constraining the lexicon. The C-bias has been consistently found in Dutch, where the number of Cs and *Vs* is comparable (Cutler et al., 2000); in Spanish, which has a considerably higher proportion of Cs than *Vs* (Cutler et al., 2000; Marks et al., 2002); in Japanese, which lacks lexical stress (Cutler and Otake, 2002); and in English, which features V reduction and lexical stress (Sharp et al., 2005; Moates and Marks, 2012). Together, these findings provide converging evidence that the C advantage is robust and cross-linguistically stable.

Beyond lexical learning, the C-Bias is evident in low-level phonological tasks such as speech segmentation and lexical decision-making. In speech segmentation tasks using artificial languages, participants identify word boundaries more easily using Cs information (Toro et al., 2008; Bonatti et al., 2005; Newman et al., 2011). In lexical decision tasks, masking Cs with white noise interferes more with lexical access than masking *Vs* (Owren and Cardillo, 2006). Such findings suggest that the asymmetry extends beyond higher-level lexical processes, reflecting a broader bias in phonological encoding.

The manifestation of C-Bias has been attributed to several key factors: 1) acoustic differences and the ratio of Cs to *Vs* (Van Ooijen, 1996); 2) universal acoustic-perceptual distinctions (Cutler et al., 2000; Nespor et al., 2003); and 3) their roles based on word position (Creel et al., 2006). Van Ooijen (1996) suggested that the higher number of Cs in most languages facilitates faster access to them during WR tasks. He also proposed that the acoustic similarity among English *Vs* leads to greater confusion, prompting listeners to rely less on V information, and that cross-language differences in C–V variability might weaken the bias where inventories are more balanced.

To test these ideas, Cutler et al. (2000) conducted WR experiments in Dutch and Spanish, languages differing in their C-to-V ratios and V distinctiveness. Both languages showed a C-Bias, with V information constraining lexical selection less effectively than Cs. These results, along with findings from other languages (Marks et al., 2002; Cutler and Otake, 2002; Moates et al., 2004), supported the universal perspective, challenging Van Ooijen’s explanations and aligning with Nespor et al.'s (2003) view. According to Nespor et al. (2003), the observed asymmetry arises from inherent acoustic and perceptual differences: Cs are processed more categorically, playing a pivotal role in lexical encoding, while *Vs* are more stable spectrally and crucial for prosodic and morphosyntactic processing. This universal perspective has been reinforced by numerous studies (Bonatti et al., 2005; Mehler et al., 2006; Escudero et al., 2016).

However, developmental research indicates that the C-Bias may not be present from the earliest stages of language acquisition. Studies with infants show that the bias does not occur uniformly across development (Bouchon et al., 2014), and V biases have even been reported (Curtin et al., 2009; Escudero et al., 2014; Højen and Nazzi, 2015). In contrast, a C-Bias has been observed in infants learning French, Italian, and British English between 8 and 30 months of age (Von Holzen et al., 2018; Poltrock and Nazzi, 2015; Hochmann et al., 2011; Mani and Plunkett, 2010). These findings suggest that the bias is not innate but emerges through linguistic experience, developing as infants gain exposure to the phonotactic and lexical regularities of their language (Keidel et al., 2007; Floccia et al., 2013).

Interestingly, Luche et al. (2013) observed a V advantage in a specific subset of stimuli—iambic CVCV words in both English and French—within an auditory lexical decision task. Although these findings initially seemed to contradict the C advantage, the authors explained the effect as resulting from rhyme preservation: in those words, the V tier coincided with the entire rhyme, producing facilitation unrelated to V processing per se. When rhyme overlaps were controlled in a subsequent experiment, the C-bias re-emerged. Thus, despite local V facilitation effects, their results ultimately supported Nespor et al.’s proposal of a universal C-bias in lexical processing, while indicating that rhyme information can temporarily override this pattern in specific phonological contexts.

In contrast, Creel et al. (2006) proposed a context-dependent view, suggesting that the C-Bias may vary with word position. They argued that Cs′ prominence in initial word positions, particularly in English, drives the effect. To test this, they conducted a WL experiment using VC(C) VC pseudowords that avoided initial Cs. No C-advantage was observed, indicating that consonantal influence may diminish in final positions while *Vs* gain prominence. However, the lack of replication and the use of synthesized speech limited generalizability. Nonetheless, these studies collectively suggest that contextual and phonotactic factors might interact with the C-Bias under specific linguistic conditions.

Building on this idea, more recent cross-linguistic studies have questioned whether the C-Bias truly represents a universal principle or is shaped by language-specific and cognitive factors. In Cantonese, where tonal information plays a significant role in lexical representation, no C advantage was found in WL tasks (Poltrock et al., 2018). Instead, Gómez et al. (2017) observed a V and tone advantage in an artificial-language segmentation task. According to Poltrock et al. (2018), the absence of the C-Bias could be due to the presence of an additional element that provides information for lexical encoding, leading to a “division of labor” that modifies the weight of consonantal cues. Additionally, Escudero et al. (2022) demonstrated that C-Bias effects in second-language learning vary depending on the learner’s native language, indicating that the bias may also reflect individual and L1-based differences.

Although strong evidence for a C-Bias has been reported across languages, most of the research has relied on cross-linguistic comparisons, whereas very few studies have examined its variability within a single language. Cross-linguistic approaches have provided valuable insights into the generality of the phenomenon, but they are insufficient to explain how phonotactic structure modulates the bias. Moreover, the few studies that have explored within-language variation, most notably Luche et al. (2013) and Creel et al. (2006), are precisely those that have reported divergent or context-dependent results. This pattern suggests that the C-Bias may be sensitive to specific phonological conditions within a language, yet this dimension has received little systematic attention. Investigating the C-Bias within a single language therefore provides a more controlled framework for isolating the phonotactic factors that modulate it. For example, segment position within the syllable or factors enhancing V discriminability could influence the bias (Creel et al., 2006; Gómez et al., 2017; Højen and Nazzi, 2015). Studying the phenomenon within one linguistic system also minimizes cross-linguistic confounds such as inventory size or stress patterns, allowing a more precise assessment of phonotactic influences.

In summary, the existing literature reveals three key patterns. First, the C-Bias has been consistently documented across multiple languages and tasks, supporting its characterization as a robust phenomenon in phonological processing. Second, however, recent evidence from tonal languages such as Cantonese and developmental studies suggests that the bias is not universal but rather emerges through linguistic experience and can be modulated by additional phonological cues. Third, the few studies that have examined within-language variation—manipulating factors such as word position or syllabic structure—are precisely those that have reported context-dependent or divergent results, pointing to the need for systematic investigation of how phonotactic properties shape the bias within a single language. Despite these advances, no study to date has directly tested whether syllabic structure modulates the C-Bias within a language whose V system is both acoustically distinctive and subject to duration-dependent variation.

To address these gaps, the present study focuses on Spanish, aiming to investigate how phonotactic properties shape C and V processing. Spanish is ideal for examining this issue due to its phonemic inventory, with 20 Cs compared to just 5 *Vs* (Maddieson, 1984). Despite this, Spanish has a simple, symmetrical, and stable V system (Correa, 2017). The five *Vs* are acoustically distinct, making it easier for native speakers to discriminate *Vs* from one another than to distinguish Cs (Cutler et al., 2000; Marks et al., 2002). Phonetic distance has been shown to play a critical role in word learning. For instance, Curtin et al. (2009) demonstrated that minimal pairs with greater interphonemic distance between *Vs* are learned more effectively in minimal pair learning paradigms. This suggests that the acoustic distinctiveness and greater interphonemic distance of Spanish *Vs* may facilitate word learning. Additionally, V duration in Spanish varies systematically with syllabic structure, a phenomenon known as “polysyllabic shortening” (Santos et al., 1998). As the number of syllables in a word increases, V duration decreases, while in shorter words, *Vs* are proportionally longer (Cuenca, 1996). These characteristics of the Spanish V system—its acoustic distinctiveness, interphonemic distance, and duration patterns—could reduce the likelihood of a C-Bias.

Building on these phonotactic features and V-duration dynamics, the present study tests whether the manifestation of the C-Bias is modulated by phonotactic structure. By comparing monosyllabic and disyllabic words in a Cross-Situational Word Learning (CSWL) task, we aim to evaluate the stability of the C-Bias across different lexical contexts within the same language. This approach allows us to determine whether the C-Bias reflects a universal phonological constraint or a language-specific adaptation shaped by Spanish phonotactics.

## 2 Materials and methods

### 2.1 Participants

Ninety students from the University of Málaga participated voluntarily in exchange for academic credit. All participants completed a linguistic background questionnaire at the start of the session, which collected demographic and linguistic information, including languages spoken, knowledge of any language other than Spanish, and self-reported proficiency level using the Common European Framework of Reference (A1–C2). Of these, 87 were native Spanish speakers; three were native speakers of other languages and were excluded from analyses. All remaining participants were born in an Andalusian province, and none reported being bilingual; while some indicated knowledge of English as a foreign language, their self-reported proficiency levels were low. Additionally, nine participants were removed due to hardware-related issues during the task, three did not complete the task, and one was excluded due to a dyslexia diagnosis. The final sample consisted of 77 native Spanish-speaking participants (66 females; 11 males; M age = 22.12 years, SD = 3.15).

### 2.2 Stimuli

The recordings for the experiment were recorded by a 25-year-old speech therapist, a native speaker of the Andalusian dialect of Spain, selected to match the regional background of the participants. The speaker recorded 16 pseudowords in two syllabic structures: 8 monosyllabic pseudowords (CVC) structure and 8 disyllabic pseudowords (CVCV) structure. In each structure, 4 pseudowords differed in their initial C (GAR, SAR, FAR, TAR for CVC and JAFE, PAFE, SAFE, CHAFE for CVCV), and 4 differed in their initial V (JES, JIS, JOS, JUS for CVC and FAFO, FEFO, FIFO, FUFO for CVCV)” (see Table 1).

**Table 1:** Pseudowords and visual referents used in each structure of minimal vowel pairs.

Structure CVC			
JES	JIS	JOS	JUS
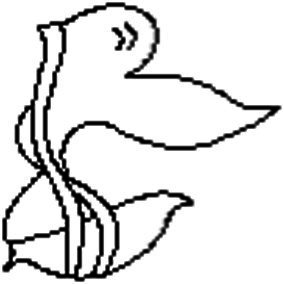	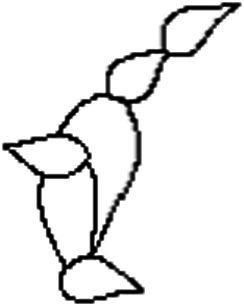	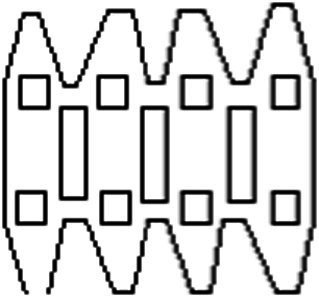	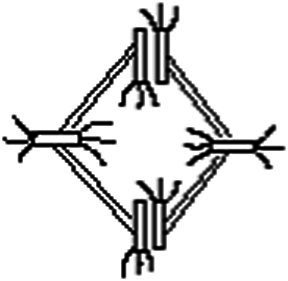

Structure CVCV			
FEFO	FIFO	FAFO	FUFO
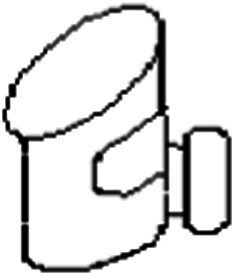	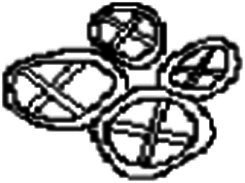	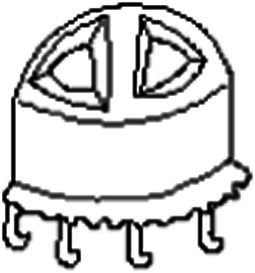	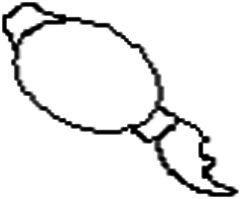

#### 2.2.1 Minimal pairs of vowels (vowelMP)

Considering the phenomenon of polysyllabic shortening (Cuenca, 1996), words with CVC structure were labeled as a condition that favors *Vs*, while the CVCV structure was labeled as a condition that does not favor Vs. Other phonological phenomena that could alter *Vs* were controlled for, such as stress or coarticulation of nearby Cs. To achieve this, all *Vs* forming the minimal pair in both CVC and CVCV structures were stressed. To control coarticulation phenomena, all Cs preceding and following the *Vs* were fricative Cs.

Using the PRAAT program (Boersma and Weenink, 2001), audio analysis was conducted to verify the parameters of the *Vs* in the two structures. The analyzed parameters were fundamental frequency (F0), first formant (F1), second formant (F2), intensity (I), total word duration (Dt), and duration of each V (Dv). Student’s *t*-test was performed between the parameters of CVC and CVCV words. The analysis revealed a significant difference only in the Dv parameter: the *Vs* of CVC words had a significantly longer duration than the *Vs* of CVCV words, confirming the effect of the polysyllabic reduction phenomenon. This analysis ensured that the only parameter that varied between the two sets of pseudowords corresponded to the duration of the first V.

#### 2.2.2 Minimal pairs of consonants (consMP)

For the creation of consonant minimal pairs (consMP), interphonemic distance was considered to avoid creating overly similar contrasts. According to Lecours and Lhermitte (1969), the more distinctive features two phonemes share, the smaller their interphonemic distance and, consequently, the greater their perceptual similarity. Following this criterion, Cs differing by two distinctive features were selected whenever possible (Jakobson and Halle, 1967; Lecours and Lhermitte, 1969).

In the CVC structure, this procedure yielded three pairs differing in two distinctive features, two pairs differing in three features, and one pair differing in a single feature. In the CVCV structure, five pairs differed in two features and one in a single feature. The corresponding distinctive features for each C pair are listed in Table 1.

It was not possible to keep the contrasts identical across the two syllabic structures, as doing so would have produced existing Spanish words. Because our primary criterion was to use pseudowords to avoid lexical activation effects, slight variations in the consonantal contrasts were necessary to ensure that all stimuli remained non-lexical.

Each pseudoword was randomly paired with a novel visual referent (see Table 2), consisting of black and white drawings of novel objects previously used in CSWL studies (e.g., Tuninetti et al., 2020). The pseudoword-visual referent pairings were counterbalanced. Pseudoword pairings created three matching conditions: minimal pairs of consonants (consMP), minimal pairs of vowels (vowelMP), and non-minimal pairs (nonMP), without phonological similarity. For example, in the monosyllabic CVC structure, a nonMP could be JIS-TAR, a consMP could be SAR-TAR, and vowelMP pair could be JIS-JOS. Similarly, in the disyllabic CVCV structure, a nonMP could be FEFO-SAFE, a consMP could be JAFE-PAFE, and vowelMP could be FAFO-FEFO (Table 3).

**Table 2:** Pseudoword stimuli, transcriptions, and phonological contrasts across conditions.

Conditions	Pair	Transcription	Contrasts
Consonants CVC	Gar – Sar	/ˈɡaɾ/ – /ˈsaɾ/	voiced velar plosive /ɡ/ vs. voiceless alveolar fricative /s/.
	Gar – Far	/ˈɡaɾ/ – /ˈfaɾ/	voiced velar plosive /ɡ/ vs. voiceless labiodental fricative /f/.
	Gar – Tar	/ˈɡaɾ/ – /ˈtaɾ/	voiced velar plosive /ɡ/ vs. voiceless dental plosive /t/.
	Sar – Far	/ˈsaɾ/ – /ˈfaɾ/	voiceless alveolar fricative /s/ vs. voiceless labiodental fricative /f/.
	Sar – Tar	/ˈsaɾ/ – /ˈtaɾ/	voiceless alveolar fricative /s/ vs. voiceless dental plosive /t/.
	Far – Tar	/ˈfaɾ/ – /ˈtaɾ/	voiceless labiodental fricative /f/ vs. voiceless dental plosive /t/.
Vowels CVC	Jes – Jis	/ˈxes/ – /ˈxis/	mid-close front vowel /e/ vs. close front vowel /i/.
	Jes – Jos	/ˈxes/ – /ˈxos/	mid-close front vowel /e/ vs. mid-close back vowel /o/.
	Jes – Jus	/ˈxes/ – /ˈxus/	mid-close front vowel /e/ vs. close back vowel /u/.
	Jis – Jos	/ˈxis/ – /ˈxos/	close front vowel /i/ vs. mid-close back vowel /o/.
	Jis – Jus	/ˈxis/ – /ˈxus/	close front vowel /i/ vs. close back vowel /u/.
	Jos – Jus	/ˈxos/ – /ˈxus/	mid-close back vowel /o/ vs. close back vowel /u/.
Consonants CVCV	Jafe – Pafe	/ˈxa.fe/ – /ˈpa.fe/	voiceless velar fricative /x/ vs. voiceless bilabial plosive /p/.
	Jafe – Safe	/ˈxa.fe/ – /ˈsa.fe/	voiceless velar fricative /x/ vs. voiceless alveolar fricative /s/.
	Jafe – Chafe	/ˈxa.fe/ – /ˈtʃa.fe/	voiceless velar fricative /x/ vs. voiceless palato-alveolar affricate /tʃ/.
	Pafe – Safe	/ˈpa.fe/ – /ˈsa.fe/	voiceless bilabial plosive /p/ vs. voiceless alveolar fricative /s/.
	Pafe – Chafe	/ˈpa.fe/ – /ˈtʃa.fe/	voiceless bilabial plosive /p/ vs. voiceless postalveolar affricate /tʃ/
	Safe – Chafe	/ˈsa.fe/ – /ˈtʃa.fe/	voiceless alveolar fricative /s/ vs. voiceless palato-alveolar affricate /tʃ/.
Vowels CVCV	Fafo – Fefo	/ˈfa.fo/ – /ˈfe.fo/	open back vowel /a/ vs. mid-close front vowel /e/.
	Fafo – Fifo	/ˈfa.fo/ – /ˈfi.fo/	open back vowel /a/ vs. close front vowel /i/.
	Fafo – Fufo	/ˈfa.fo/ – /ˈfu.fo/	open back vowel /a/ vs. close back vowel /u/.
	Fefo – Fifo	/ˈfe.fo/ – /ˈfi.fo/	mid-close front vowel /e/ vs. close front vowel /i/.
	Fefo – Fufo	/ˈfe.fo/ – /ˈfu.fo/	mid-close front vowel /e/ vs. close back vowel /u/.
	Fifo – Fufo	/ˈfi.fo/ – /ˈfu.fo/	close front vowel /i/ vs. close back vowel /u/.

**Table 3:** fixed effects from the generalized linear mixed-effects model predicting learning accuracy.

Predictor	*β*	SE	95% CI	Odds ratio	*p*
Intercept (consMP · CVC)	2.37	0.27	[1.84, 2.90]	10.70	<0.001
Main effects					
Pair Type: NonMP vs. consMP	0.69	0.17	[0.36, 1.02]	2.00	<0.001
Pair Type: VowelMP vs. consMP	0.47	0.22	[0.04, 0.90]	1.60	0.030
Structure: CVCV vs. CVC	−0.05	0.29	[−0.62, 0.52]	0.95	0.862
Interaction					
NonMP × CVCV	−0.43	0.22	[−0.86, 0.00]	0.65	0.054
VowelMP × CVCV	−0.92	0.28	[−1.47, −0.37]	0.40	0.001

Summary of the fixed effects from the final binomial generalized linear mixed-effects model. The reference level for Pair Type is consonant minimal pairs (consMP), and for Structure is CVC. The dependent variable was accuracy (correct/incorrect). *β* represents the estimated log-odds coefficient, SE is the standard error, 95% CI is the 95% Confidence Interval for the coefficient, and Odds Ratio is the exponentiated coefficient (e^β). *p*-values are based on Wald z-tests. The model’s R-squared values were Marginal R^2^ = 0.026 and Conditional R^2^ = 0.339.

### 2.3 Procedure

The experiment comprised two blocks: one containing eight monosyllabic (CVC) stimuli and another with eight disyllabic (CVCV) stimuli. Each block consisted of a learning phase and a testing phase, and the order of presentation of the two blocks was randomized across participants.

The task was programmed and presented using E-Prime 3.0 (Psychology Software Tools, Pittsburgh, PA, USA). The sessions were conducted in groups of up to 10 participants, each seated at an individual workstation acoustically and visually isolated from the others to prevent distraction or interference. Participants were seated approximately 60 cm from a 17-inch monitor in a quiet testing room. Auditory stimuli were delivered through high-quality headphones, and responses were made using a standard keyboard.

Participants were instructed to pay close attention to the images and the words they would hear. They were not informed that the words corresponded to the names of the images, nor were they explicitly asked to associate the words with the images.

During the testing phase (described in detail in Section 2.3.2), participants selected the image corresponding to each auditory pseudoword via key press. Response accuracy and reaction times were automatically recorded by E-Prime 3.0. The entire experimental session lasted approximately 15 min.

#### 2.3.1 Learning phase

**Figure 1:**
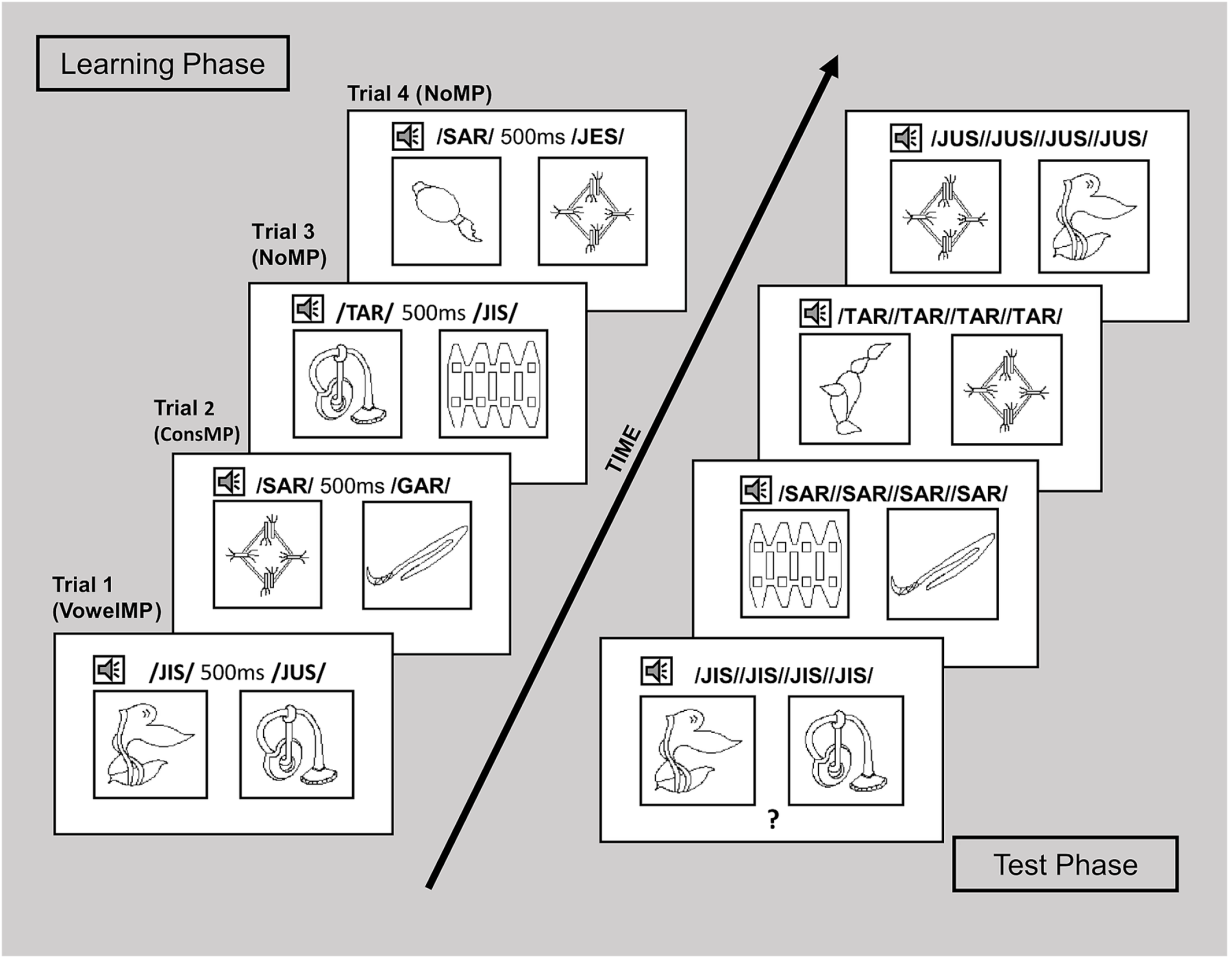
Examples of learning and test trials.

Each block’s learning phase comprised 36 trials, during which participants were exposed to each word-visual referent combination nine times. A trial involved displaying two images of objects on the screen and playing two corresponding pseudoword recordings (see Figure 1). Each trial featured only one of the three pseudoword pairing conditions discussed earlier (nonMP; consMP or vowelMP). Thus, each block’s learning phase (CVC block and CVCV block) comprised a total of 36 pairs: 24 nonMP, 6 consMP, and 6 vowelMP.

In each learning trial, two of the eight visual referents were shown against a white background with a resolution of 800 × 600 pixels. The top-left corner of the left image was 20 × 163 pixels, and the top-left corner of the right image was at 500 × 163 pixels. Each image measured 280 × 274 pixels. The pseudowords associated with the images were played 500 ms after the images appeared on the screen, so that each image was named once, either from left to right or vice versa, with a 500 ms interval between the spoken words. Participants were unaware of the order in which visual referents would be named. Learning trials were presented randomly for each participant and controlled to ensure that each visual referent combination appeared at least once and no more than twice. If the same combination was repeated, the left-to-right assignments were reversed to ensure that participants did not see the same visual association more than once. The appearance of an image on the left or right side was balanced so that half of the word associations were shown five times on the left and four times on the right, while the other half were presented four times on the left and five times on the right.

#### 2.3.2 Testing phase

The testing phase began immediately after the learning stage. Participants completed a forced-choice task where they heard an auditory pseudoword and selected, via key press, the image (left or right) corresponding to the pseudoword. Trials used the same novel object images as the learning phase, with random left–right assignments to ensure consistency across participants. Responses were organized by trial type, based on the phonological overlap between the pseudowords associated with the images: nonMP (e.g., in CVC: FAR-JES; in CVCV: CHAFE-FIFO); consMP (e.g., in CVC: FAR-TAR; in CVCV: CHAFE-PAFE); and vowelMP (e.g., in CVC: JES-JIS; in CVCV: FAFO-FIFO). In each trial, after the images appeared for 500 ms, the pseudoword was played four times before participants responded (see Figure 1). Each word-image pair was presented four to five times. As in the learning phase, trials included 24 nonMP, 6 vowelMP and 6 consMP. The trial order was randomized for each participant.

## 3 Results

**Figure 2:**
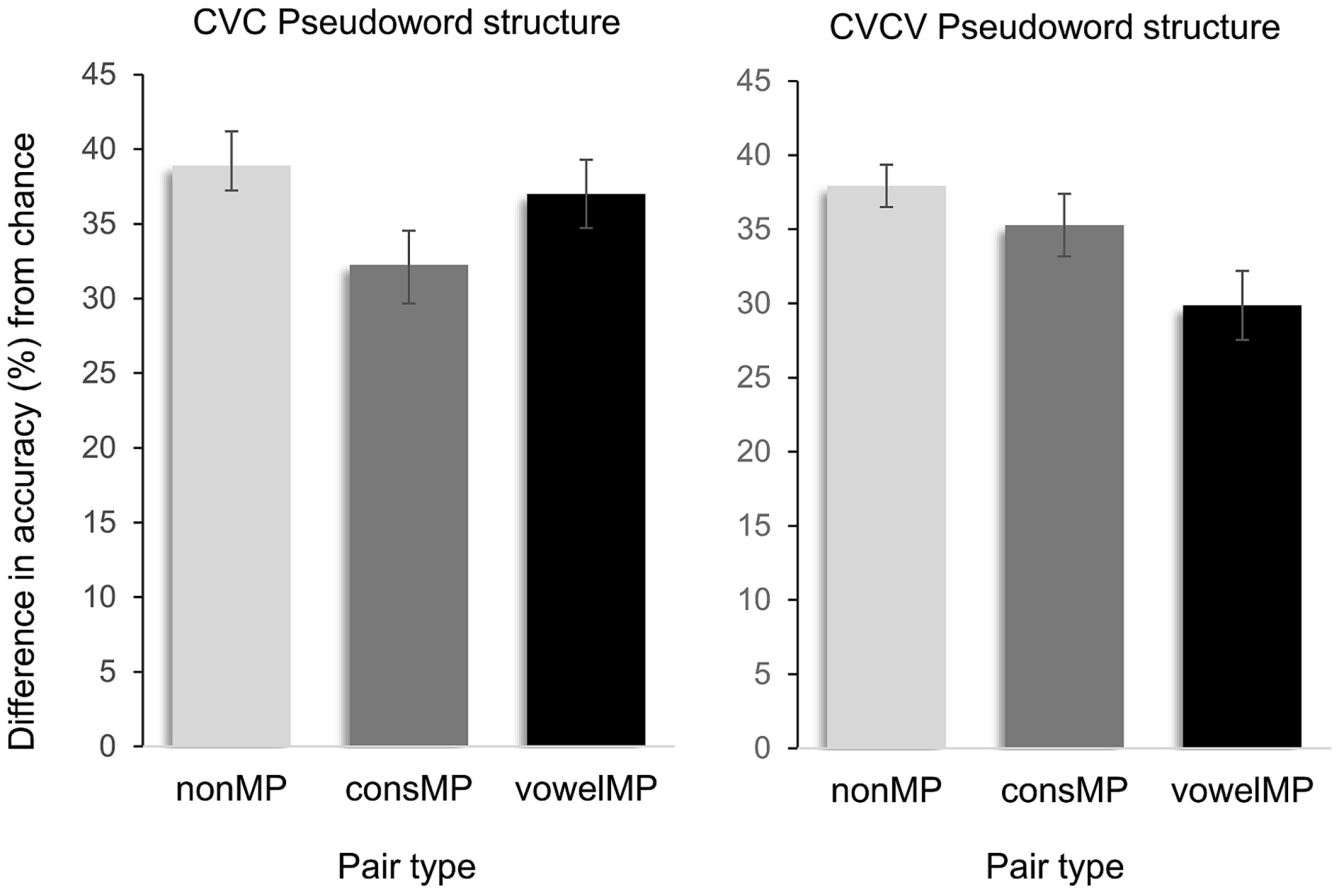
Difference in percent accurate identification of auditory words and their visual referents from chance after cross-situational training, in CVC and CVCV pseudoword structures.

Firstly, to verify if the experiment worked in the same way as in previous studies in other languages (Mulak et al., 2019; Escudero et al., 2016), we conducted an initial analysis. This analysis aimed to determine whether learners could infer pseudoword-object pairings via cross-situational statistical learning. Therefore, we conducted one-sample *t*-tests comparing participants’ accuracy against chance (0.50) for each pair type and syllabic structure. As shown in Figure 2, performance was significantly above chance across all conditions. In the CVC structure, accuracy reached 88.99% for nonMPs [nonMPs; SEM = 1.67, *t*(76) = 53.42, *p <* 0.001], 82.25% for consMPs [consMPs; SEM = 2.58, *t*(76) = 31.85, *p <* 0.001], and 87.01% for vowelMPs [vowelMPs; SEM = 2.29, *t*(76) = 37.97, *p <* 0.001]. In the CVCV structure, performance remained high—nonMPs: 88.00% [SEM = 1.43, *t*(76) = 61.61, *p <* 0.001]; consMPs: 85.25% [SEM = 2.11, *t*(76) = 40.42, *p <* 0.001]; vowelMPs: 79.86% [SEM = 2.33, *t*(76) = 34.25, *p <* 0.001]. These results confirm that participants successfully learned word-object mappings in all pair and structure conditions.

To assess the influence of phonological similarity (Pair Type: nonMP, consMP, vowelMP) and syllabic structure (Structure: CVC, CVCV) on learning accuracy, data were analysed with a binomial mixed-effects model (GLMM; Baayen, 2012; Jaeger, 2008) in R (lme4: Bates et al., 2015; sjPlot: Lüdecke, 2023). To determine the most appropriate random effects structure, we followed a stepwise model-building approach. Since both Pair Type and Structure were within-subjects factors, we tested for the inclusion of by-subject random slopes.

A model including by-subject random slopes for Structure provided a significantly better fit to the data than a model with only random intercepts for subjects and items [χ^2^(3) = 109.72, *p <* 0.001]. We also attempted to include random slopes for Pair Type and its interaction with Structure, but this resulted in a singular fit, indicating over-parameterization.

Therefore, the final, most parsimonious model included fixed effects for Pair Type (reference: consMP), Structure (reference: CVC), and their interaction. The random effects structure consisted of by-subject random intercepts and by-subject random slopes for Structure, as well as by-item random intercepts. The model’s R-squared values were Marginal R^2^ = 0.026 and Conditional R^2^ = 0.339 (Nakagawa et al., 2017).

**Figure 3:**
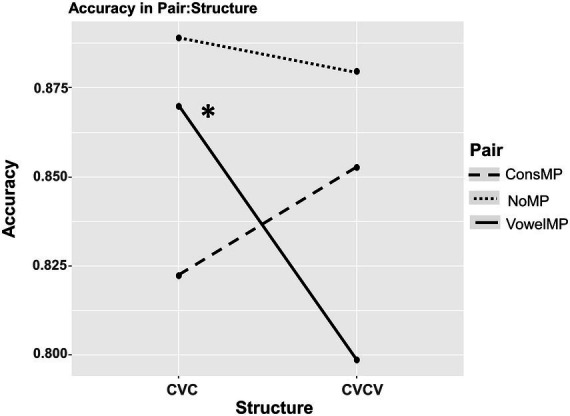
Interaction of pair type and structure in accuracy. *Note*: Interaction between pair type and structure on word learning accuracy. Proportion of correct responses is plotted by pair type (nonMP, vowelMP, consMP) across syllabic structures (CVC, CVCV). The figure illustrates a significant interaction, showing that accuracy for vowel minimal pairs declined notably in the CVCV condition, while performance for consonant minimal pairs was particularly low in the CVC condition. Non-minimal pairs showed consistently high accuracy across both structures.

Critically, these main effects were qualified by a significant interaction between Pair Type and Structure, as visualized in Figure 3. The interaction was driven by the performance on vowelMPs across structures (PairVowelMP × StructureCVCV: *β* = −0.92, *p <* 0.001). Planned comparisons confirmed this pattern:

- In the CVC structure, learners showed a vowel bias: accuracy for vowelMPs was higher than for consMPs (OR = 1.61), and statistically indistinguishable from nonMPs (*β* = 0.22, *p* = 0.208).
- In the CVCV structure, this pattern shifted: accuracy for vowelMPs declined significantly compared to nonMPs (*β* = −0.72, *p* = 0.003), but vowelMPs and consMPs did not differ significantly from each other (*β* = 0.46, *p* = 0.192). This suggests a partial C-bias, in which the previous V advantage disappears, but consMPs do not become significantly easier to learn than vowelMPs.

These findings indicate that phonological bias is not fixed and is modulated by the structural properties of the words being learned. Learners’ reliance on V versus C cues appears context-dependent, showing a V advantage in simpler structures (CVC), and a reduction or reversal of this effect in more complex forms (CVCV), consistent with a partial shift toward consonantal encoding under increased processing demands.

## 4 Discussion

In this study, we aimed to investigate the manifestation of the C-Bias in Spanish lexical processing, focusing on how phonotactic and acoustic-contextual factors influence its presence. While we hypothesized the existence of a C-advantage, we proposed that this bias would be modulated by the phonotactic complexity of Spanish, particularly its syllabic structure.

Participants successfully learned pseudoword-object associations through cross-situational statistical learning, confirming the reliability of our task, as demonstrated in previous studies (Escudero et al., 2016). However, the manifestation of phonological bias was not consistent across different syllabic structures. In CVC pseudowords, consMPs were learned significantly less accurately than nonMPs, while vowelMPs performed similarly to nonMPs and significantly better than consMPs, revealing and reinforcing a V-Bias. In contrast, in CVCV pseudowords, consMPs were learned as accurately as nonMPs, but their performance did not differ significantly from that of vowelMPs. This pattern suggests a partial C-bias: although consMPs were learned as well as nonMPs and vowelMPs showed significantly lower accuracy than nonMPs, no significant difference was found between consMPs and vowelMPs. These findings indicate that phonological bias in word learning is modulated by the syllabic structure of the input, with V cues being more effective in simpler word forms and less informative under greater structural complexity.

The V bias observed in CVC words likely resulted from V lengthening, which enhanced V discriminability and reduced reliance on Cs for lexical distinction. In contrast, in CVCV words, the V-bias weakened, showing a trend toward better performance for consMP. This shift can be explained by the phenomenon of polysyllabic shortening in Spanish (Cuenca, 1996), whereby V duration decreases in longer word forms, diminishing the informativeness of V cues and increasing the relative contribution of Cs. This pattern supports the idea that polysyllabic shortening primarily affects *Vs*, accounting for the differential performance of vowelMP across structures. Although Spanish *Vs* are acoustically salient and widely spaced in the phonetic space (Curtin et al., 2009), these features alone do not suffice to maintain their advantage under conditions where duration is reduced. Consequently, while Cs did not become significantly more informative, they showed a relative increase in discriminative weight when V cues were weakened by structural constraints.

This outcome contrasts with the two previous studies conducted in Spanish using word reconstruction tasks (Cutler et al., 2000; Marks et al., 2002), both of which reported a C-bias. However, these studies differ methodologically from ours: they used small participant samples (20 participants in Marks et al., 2002) and employed real bi- and trisyllabic words, rather than monosyllables—the structure where we observed a V-bias and which, unlike longer forms, is not affected by polysyllabic shortening. Interestingly, the structure in which our data revealed a mild trend toward consonantal facilitation (disyllables) corresponds to the same syllabic configurations where a C-Bias has been found in Spanish (bi- and trisyllables). This alignment reinforces our proposal that Cs become more discriminative when V cues lose prominence due to polysyllabic shortening. Future research should test whether a similar V-bias emerges for monosyllabic items in a word reconstruction task, to determine whether the phenomenon depends on phonotactic structure or on task-specific processing demands.

In addition to the increased V duration in CVC words, another factor that could contribute to the V bias is the lower frequency of monosyllabic words in Spanish and their reduced phonological neighborhood density. Lexical frequency and neighborhood density are both key factors in lexical learning and processing (Garlock et al., 2001). In Spanish, disyllabic words are far more frequent than monosyllabic ones (Quilis, 1993), suggesting that the use of less frequent monosyllabic words with sparse phonological neighborhoods may reduce the reliance on consonantal cues to differentiate new words from those already represented in the lexicon. Beyond phonetic explanations, lexical-distributional properties may therefore also contribute to the observed asymmetries.

Taken together, our findings sharpen—and complicate—current explanations of the C-Bias. Although many studies treat the bias as a universal perceptual asymmetry, growing evidence points to a dynamic phenomenon shaped by the acoustic-contextual properties of each language. Early accounts highlighted inventory size or V similarity, arguing that the sheer number of Cs (Van Ooijen, 1996) or their typical word-initial position (Creel et al., 2006) drives the advantage. More recently, the “division-of-labour” proposal (Poltrock et al., 2018) showed that additional phonological cues—such as tone in Cantonese—can redistribute perceptual weight away from Cs. Curtin et al.’s (2009) Phonetic Distance Hypothesis complements this account by predicting that Spanish *Vs*, which are acoustically well spaced, attain high salience when temporal cues favour them—as in elongated monosyllables. Yet these explications alone cannot explain why the bias reverses in CVCV items; only a context-sensitive framework captures the full pattern.

To address these complexities, we propose the Contextual Dependence Hypothesis of Maximum Discrimination. According to this hypothesis, the discriminability of linguistic segments is shaped not only by phonetic contrasts but also by a range of contextual factors, including syllabic structure, lexical frequency, stimulus length, and the relative positioning of Cs and Vs. Our findings suggest that the phonological processing system is flexible, adapting to these factors by prioritizing the most discriminable segments depending on the context. For instance, in our study, *Vs* were more readily discriminated in monosyllabic CVC structures, whereas Cs regained primacy in disyllables—an illustration of adaptive cue weighting. In line with this hypothesis, we observed substantial inter-individual variability in learning performance. The random-effects structure of the model, as well as the large discrepancy between marginal and conditional R^2^ values, suggest that listeners differ considerably in how efficiently they learn each pattern. Importantly, these individual differences do not merely reflect varying degrees of C-bias; rather, they point to flexible segmental weighting strategies that are dynamically modulated by both the statistical properties of the input and the learner’s own processing tendencies or cognitive profile.

The comparison with the results of Escudero et al. (2016) provides additional insight into the variability of the C-Bias across languages. In their study, English speakers performing the same CSWL task with CVC items exhibited a robust C-Bias, with significantly lower accuracy for vowelMPs than for consMPs or nonMPs. In contrast, our Spanish speakers showed the opposite pattern in the same structure. This cross-linguistic divergence can be interpreted through the Linguistic Perception (LP) model (Escudero, 2005) and its extension, the L2LP model (Van Leussen and Escudero, 2015; Escudero et al., 2022; Escudero and Yazawa, 2024), which propose that native phonological categories shape how segmental contrasts are perceived and processed. In English, the V inventory is large and contains several acoustically proximal pairs (e.g., /i/−/ɪ/, /u/−/ʊ/), which increases perceptual confusability and reduces the reliability of *Vs* as lexical cues, thereby favoring consonantal information for lexical discrimination. The contrasting properties of the Spanish V system, discussed above, produce the opposite weighting. Our findings thus extend the predictions of these models beyond individual phoneme perception to the level of segment class dominance: native phonological properties not only shape how specific contrasts are processed but also determine whether Cs or *Vs* as a class carry greater weight in lexical discrimination. These results are consistent with previous findings in languages where additional phonological cues redistribute perceptual weight away from Cs (Poltrock et al., 2018; Gómez et al., 2017).

While our findings offer valuable insights, some limitations remain. The use of isolated pseudowords does not fully capture the complexity of natural language acquisition, which may constrain the generalizability of our results. In connected speech, prosodic factors such as lexical stress modulate V duration and spectral quality, potentially enhancing or diminishing V salience depending on stress position, particularly relevant consideration given that our findings suggest V discriminability is sensitive to temporal cues. Moreover, coarticulatory processes in continuous speech alter the acoustic realization of both Cs and *Vs* in ways that do not occur in isolated production, which could affect segment distinctiveness differently across CVC and CVCV structures, potentially reshaping the weighting patterns we observed. Furthermore, embedding words in sentential contexts introduces top-down lexical and syntactic information that listeners use to constrain word recognition, which may interact with the bottom-up phonetic cues examined in this study. For these reasons, future research should test whether the syllable-dependent biases reported here are maintained, attenuated, or amplified when stimuli are presented within sentential frames. Further studies should also orthogonally vary neighborhood density, test other segmental frames (e.g., VCV), and examine languages with extensive V reduction. Eye-tracking or reaction time measures could additionally reveal subtler cue-weighting dynamics that accuracy data alone may not capture.

Beyond these constraints, our results raise potential implications for models of language learning and, more tentatively, for educational practice. The observation that V cues can outweigh consonantal ones in Spanish suggests that the relative informativeness of segmental classes is not fixed but shaped by the phonotactic and phonetic properties of each language. It is therefore plausible that, in languages with highly distinctive and temporally robust V systems, instructional approaches that draw learners’ attention to vocalic contrasts might facilitate certain aspects of vocabulary learning. However, such applications remain speculative and should be directly evaluated in intervention studies before informing concrete teaching recommendations.

In summary, the present study shows that V salience in Spanish can override C dominance when phonotactic conditions favour *Vs*, whereas Cs tend to regain relevance when those conditions reduce the informativeness of V cues. These findings support a view of phonological processing as flexible and context-sensitive, with listeners dynamically weighting segmental information to maximise discrimination within the phonotactic ecology of their language.

## Data Availability

The datasets presented in this study can be found in online repositories. The names of the repository/repositories and accession number(s) can be found at: https://osf.io/exq5m/?view_only=8684a237c8dc482d988d793be1f3f01a.
